# Supplementary material for: Light Cross-Linkable Marine Collagen for Coaxial Printing of a 3D Model of Neuromuscular Junction Formation
Source: Biomedicines. 2020 Dec 26;9(1):16. doi: 10.3390/biomedicines9010016 (PMC7823301; doi:10.3390/biomedicines9010016)
Supplement: Supplementary file 1 [file biomedicines-09-00016-s001.pdf]

Supplementary Information

Light Cross-linkable Marine Collagen for Coaxial Printing of a 3D Model of Neuromuscular Junction Formation

Borja Sanz[1,2], Ane Albillos Sanchez[1,2], Bonnie Tangey[1], Kerry Gilmore[1], Zhilian Yue[1], Xiao Liu[1], Gordon Wallace[1]*

(1) ARC Centre of Excellence for Electromaterials Science, Intelligent Polymer Research Institute, AIIM Facility, Innovation Campus, University of Wollongong, Squires Way, Wollongong, New South Wales 2500, Australia.

(2) Current address: Department of Orthopedics, University Medical Center Utrecht, Utrecht, 3508 GA, The Netherlands.

***Corresponding Author**
E-mail: gwallace@uow.edu.au

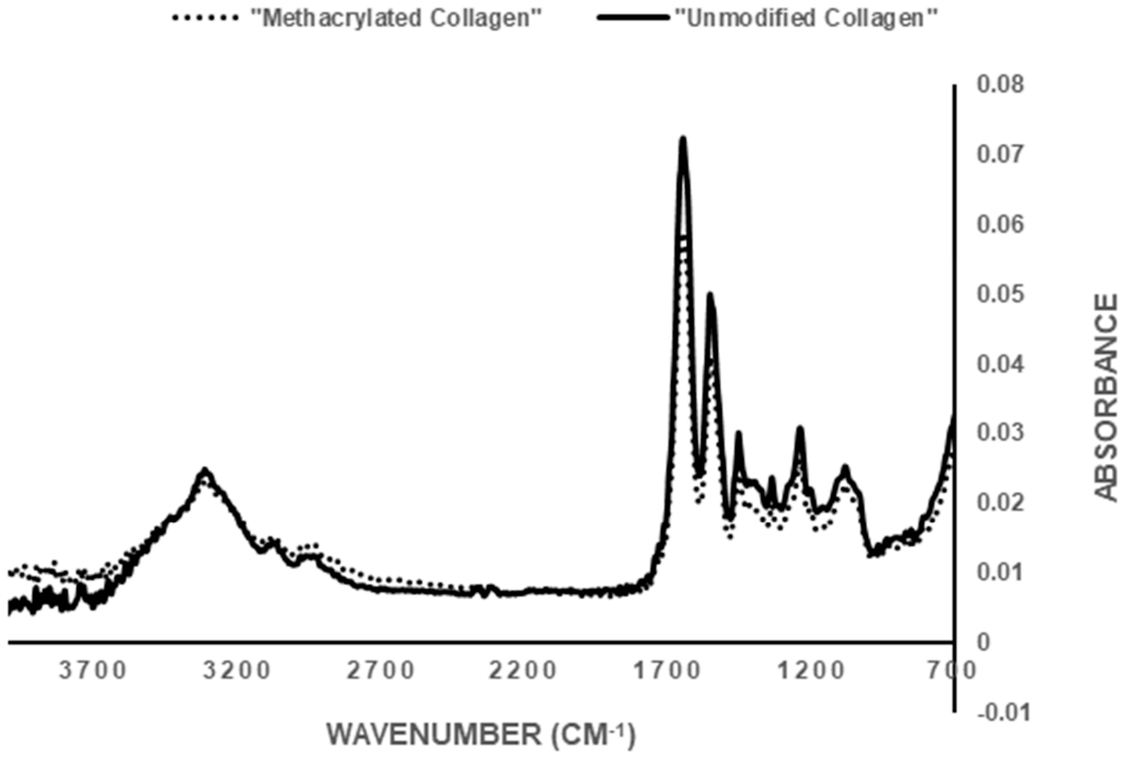

Figure 1. Comparison of FTIR spectra of extracted marine-derived collagen with (dotted line) and without (solid line) methacrylation. Samples were tested as freeze-dried foams at room temperature
